# Workplace-Based Glucose Screening for Type 2 Diabetes in French Civil Servants: Prospective Observational Cohort Study

**DOI:** 10.2196/87695

**Published:** 2025-12-22

**Authors:** Solène Petit, Florence Carrouel, Benjamin du Sartz de Vigneulles, Claude Dussart, Roger Salamon

**Affiliations:** 1 Institute of Public Health, Epidemiology and Development (ISPED), Inserm U1219, University of Bordeaux, Bordeaux, France; 2 Laboratory "Health, Systemic, Process" (P2S), UR4129, University Claude Bernard Lyon 1, 7 rue Guillaume Paradin, Lyon, 69002, France, 33 478785745; 3 Hospices Civils de Lyon, Lyon, France

**Keywords:** diabetes screening, prevention, occupational health, public service, France

## Abstract

**Background:**

Type 2 diabetes (T2D) remains one of the most underdiagnosed chronic conditions worldwide, despite its major contribution to cardiovascular and metabolic morbidity. In 2024, an estimated 589 million adults were living with diabetes globally, more than 90% of whom had T2D, and the prevalence is projected to reach 853 million by 2050. In France, approximately 4.1 million adults are affected, and nearly 1 in 4 individuals with diabetes remain undiagnosed. Therefore, early detection is essential to prevent complications. Workplace prevention strategies could improve early detection, particularly among employed adults with limited access to regular medical screening. In France, a health prevention organization has implemented a systematic glucose screening program for civil servants to identify individuals at risk of T2D or prediabetes. As the French public service includes 5.7 million workers—approximately 1 in 5 of the national workforce—this setting provides a unique opportunity to reach large, diverse, and often underserved segments of the adult population.

**Objective:**

This study aimed to assess the effectiveness of a systematic diabetes screening program as a preventive public health measure by determining the rate of newly detected diabetes cases and characterizing associated cardiometabolic risk factors within a large population of French civil servants.

**Methods:**

A retrospective observational study was conducted using data from a glucose screening program between January 2022 and February 2025. Participants with postprandial blood glucose levels >1.40 g/L were included in a follow-up cohort. Sociodemographic, clinical, and biological data were collected. Comparisons were performed using the chi-square or Fisher exact test for categorical variables and the Student *t* test (2-tailed) for continuous variables (*P*<.05). Analyses were restricted to complete cases to ensure robust comparisons.

**Results:**

Among 16,785 screened participants, 981 (5.8%) had postprandial glucose levels >1.40 g/L and 134 (0.8%) were eligible for the follow-up cohort. Participants were 59.5% (n=78) women and 40.5% (n=53) men, with a mean age of 51.3 (SD 8.9) years. Overall, 37.6% (n=50) of participants were overweight, 25.4% (n=34) were obese, 61.6% (n=77) reported insufficient physical activity, and 63.2% (n=84) had a family history of diabetes. Of the 134 eligible individuals, 70 (52.2%) completed medical follow-up, and among them, 9 (12.9%) received a confirmed diagnosis of T2D. Newly diagnosed individuals were predominantly male (n=7, 78%; *P=*.04) and more likely to be overweight or obese (n=9, 89%; *P*=.04). No significant differences in age, sex, or BMI were observed between followed and lost-to-follow-up participants.

**Conclusions:**

Systematic glucose screening in occupational or social health context identifies individuals at risk of diabetes or prediabetes and supports its integration into preventive health strategies to enhance early detection and reduce long-term complications. Larger prospective or randomized studies are warranted to confirm long-term benefits on diagnosis, care engagement, and cardiometabolic outcomes.

## Introduction

Type 2 diabetes (T2D) is one of the main public health burdens worldwide. In 2024, an estimated 11.1% of adults aged 20 to 79 years, approximately 589 million people, were living with diabetes, and more than 90% of these cases were T2D [1]. The number of adults with diabetes has quadrupled since 1990 [2] and is projected to reach 853 million by 2050 [3]. This alarming growth reflects the combined effects of rising obesity, population aging, physical inactivity, and unhealthy dietary patterns [4]. Consequently, T2D imposes substantial social, economic, and health care burdens, largely due to its cardiovascular, renal, and metabolic complications.

While prevalence continues to rise, another major challenge is the persistent underdiagnosis of T2D. Recent international evidence shows that a substantial proportion of cases remain undiagnosed, often for several years, leading to delayed intervention and advanced disease at the time of diagnosis. Global cascade-of-care analyses indicate that nearly half of the individuals living with diabetes are unaware of their condition, and fewer than 1 in 4 achieve optimal glycemic control [5,6].

In France, approximately 4.1 million adults aged 20 to 79 years were living with diabetes in 2024 [1]. The national ESTEBAN (*Étude de santé sur l'environnement, la biosurveillance, l'activité physique et la nutrition*; health study on the environment, biomonitoring, physical activity, and nutrition) survey (2014‐2016) revealed that 1.7% of French adults had undiagnosed diabetes, suggesting that nearly 1 in 4 people with diabetes were unaware of their condition at the time of the survey [7]. Despite progress in medical management, underdiagnosis remains a key determinant of delayed intervention and avoidable complications. Recent analyses from the CONSTANCES *(Cohorte Nationale de Sujets Adultes Nouveaux Constituée pour une Évaluation Systématique de la Santé*; National Cohort of New Adult Subjects Formed for Systematic Health Assessment) cohort similarly confirm that undiagnosed diabetes and prediabetes remain prevalent in France and are strongly associated with excess weight and socioeconomic vulnerability [8].

Evidence from large cohort studies and meta-analyses consistently demonstrates that early diagnosis and management of T2D—through lifestyle or pharmacological interventions—can reduce the risk of micro- and macrovascular complications by 30% to 60% and improve overall survival [9-12]. From a public health perspective, early identification through systematic screening constitutes the first step of prevention by enabling timely lifestyle counseling, glycemic control, and risk stratification.

Workplaces represent a promising setting for implementing preventive strategies, particularly within the French public sector, which employs nearly 5.7 million civil servants—about 1 in 5 workers nationwide. This population provides an ideal model for prevention studies due to its size, occupational diversity, and relatively stable follow-up potential.

Within this context, the *Union Prévention Santé pour la Fonction publique* (UROPS)—a French health promotion and prevention organization dedicated to public sector employees—has developed a national program aimed at early detection and management of chronic diseases. These preventive programs combine standardized medical assessments (postprandial glucose, BMI, and blood pressure) with lifestyle and medical questionnaires, conducted either in workplaces or regional health centers. Since 2022, this approach has been reinforced by incorporating systematic glucose testing and structured follow-up to improve the early detection of T2D and prediabetes.

Therefore, this study was conducted as a prospective observational cohort using systematically collected data from the nationwide UROPS screening program.

It was designed to address the following research question: Can a systematic workplace-based glucose screening program effectively identify previously undiagnosed T2D among French civil servants? In addition, the study aimed to characterize the cardiometabolic risk profiles of individuals presenting abnormal screening results.

## Methods

### Study Design and Setting

This study was a retrospective, observational, noncomparative study based on secondary analysis of existing data from the UROPS national database. Data were collected from January 2022 to February 2025 during standardized glucose screening campaigns implemented in the workplace that targeted public sector agents. Screening sessions were conducted at 315 sites in France.

They were implemented within workplace environments. Recruitment followed a systematic and standardized procedure. All public sector employees affiliated with UROPS and attached to an administrative site anywhere in the country (including French overseas departments and regions) received a standardized invitation issued by UROPS to participate in a scheduled screening campaign. Invitations were sent by email through workplace communication channels. Participation was voluntary; however, all public sector employees present at the screening site or a nearby auxiliary site during the scheduled period were systematically approached by the administrative services to which they belonged and encouraged to participate. Standardized posters were made available to administrations and displayed in common areas, including a registration QR code. The reception of participants and promotion of the program were ensured by UROPS representatives, who were trained and informed about the preventive objective of the initiative.

To ensure methodological consistency across all sites, screening sessions followed a unified operational protocol. Identical equipment, calibrated glucometers, structured questionnaires, and harmonized measurement procedures were used at every location. Trained nurses and technicians followed standardized procedures for participant reception, data collection, and biological measurements. Individuals presenting postprandial capillary glucose values >1.40 g/L received immediate written notification and standardized recommendations to consult a general practitioner for confirmatory laboratory testing. All follow-up procedures were identical across regions. The study was designed, analyzed, and reported in accordance with the STROBE (Strengthening the Reporting of Observational Studies in Epidemiology) guidelines [13].

### Participants

Participation in the glucose screening program was voluntary. Eligible participants were active or retired civil servants aged ≥18 years who attended a glucose screening session and provided informed consent for data use. Participants with a previous diagnosis of diabetes or incomplete files were excluded from the analysis.

### Variables

The primary outcome was the number of physician-confirmed diagnoses of diabetes following abnormal glucose screening.

Secondary variables included the following:

- Sociodemographic characteristics: age, sex, and occupational status (active or retired)
- Anthropometric and biological factors: BMI (self-reported), blood pressure, total cholesterol, high-density lipoprotein cholesterol, low-density lipoprotein cholesterol, and triglycerides
- Lifestyle factors: smoking status (current or nonsmoker), alcohol consumption, and level of physical activity (≥30 min per d vs none)
- Medical and family history: parental history of myocardial infarction or stroke, and family history of diabetes

Confirmed diabetes was defined as a diagnosis established by a physician based on laboratory tests (fasting plasma glucose ≥1.26 g/L or hemoglobin A_1c_ ≥6.5%) and documented through participant follow-up.

### Data Sources and Measurements

During each screening session, participants completed a self-administered medical questionnaire to collect sociodemographic information (age and sex); self-report anthropometric data (body weight and height); metabolic risk factors (smoking, physical activity, and alcohol use); and personal and family cardiovascular history.

Trained nurses then performed on-site biological and physiological measurements using calibrated equipment, including the following:

- Postprandial capillary glucose, measured via validated glucometers
- Blood pressure, measured using automated sphygmomanometers

Participants with postprandial capillary glucose levels >1.40 g/L were informed of their result and received a written recommendation to consult their general practitioner for confirmatory laboratory testing.

Follow-up information—including medical consultation date, laboratory results, final diagnosis, and any reported lifestyle changes—was collected several weeks later.

### Study Size

As the study aimed to analyze the totality of eligible participants screened within the observation period, no a priori sample size calculation was performed. Therefore, this study represents an exhaustive descriptive analysis of available data collected.

### Quantitative Variables

Continuous variables (eg, age, BMI, glucose, and lipid levels) were analyzed as mean (SD). BMI was categorized according to the World Health Organization criteria (underweight <18.5 kg/m^2^, normal 18.5 kg/m^2^‐24.9 kg/m^2^, overweight 25.0 kg/m^2^‐29.9 kg/m^2^, and obese ≥30.0 kg/m²) [14]. Categorical variables were expressed as numbers and percentages.

### Statistical Methods

Individuals identified with postprandial capillary glucose values >1.40 g/L (7.8 mmol/L) were considered to have abnormal screening results and were invited to undergo medical follow-up. This threshold corresponds to the value used by French occupational health authorities to indicate potential postprandial hyperglycemia and warrant confirmatory testing [15]. Group comparisons (confirmed vs nonconfirmed diabetes) were performed using the chi-square test or the Fisher exact test for categorical variables and the Student *t* test (2-tailed) for continuous variables.

Analyses were conducted using R statistical software (version 4.3.3; R Foundation for Statistical Computing). Only complete cases were included, and no data imputation was applied. A *P* value <.05 was considered statistically significant.

### Ethical Considerations

The study was approved by the Committee for the Protection of Individuals Est I (2021-A01983-38) on November 4, 2021, and complied with the Declaration of Helsinki. All participants provided written or electronic informed consent for the use of anonymized data for research purposes, in compliance with French Data Protection Authority (CNIL [*Commission Nationale de l’Informatique et des Libertés*]) regulations. They did not receive any financial or nonfinancial compensation for their participation. Data confidentiality and participant anonymity were rigorously maintained throughout the study.

## Results

### Participants

Between January 2022 and February 2025, a total of 16,785 individuals participated in the national glucose screening program across 12 regional sites in metropolitan France.

Among these, 981 participants (5.8%) had postprandial capillary glucose levels exceeding 1.40 g/L and were therefore eligible for follow-up.

**Figure 1.**
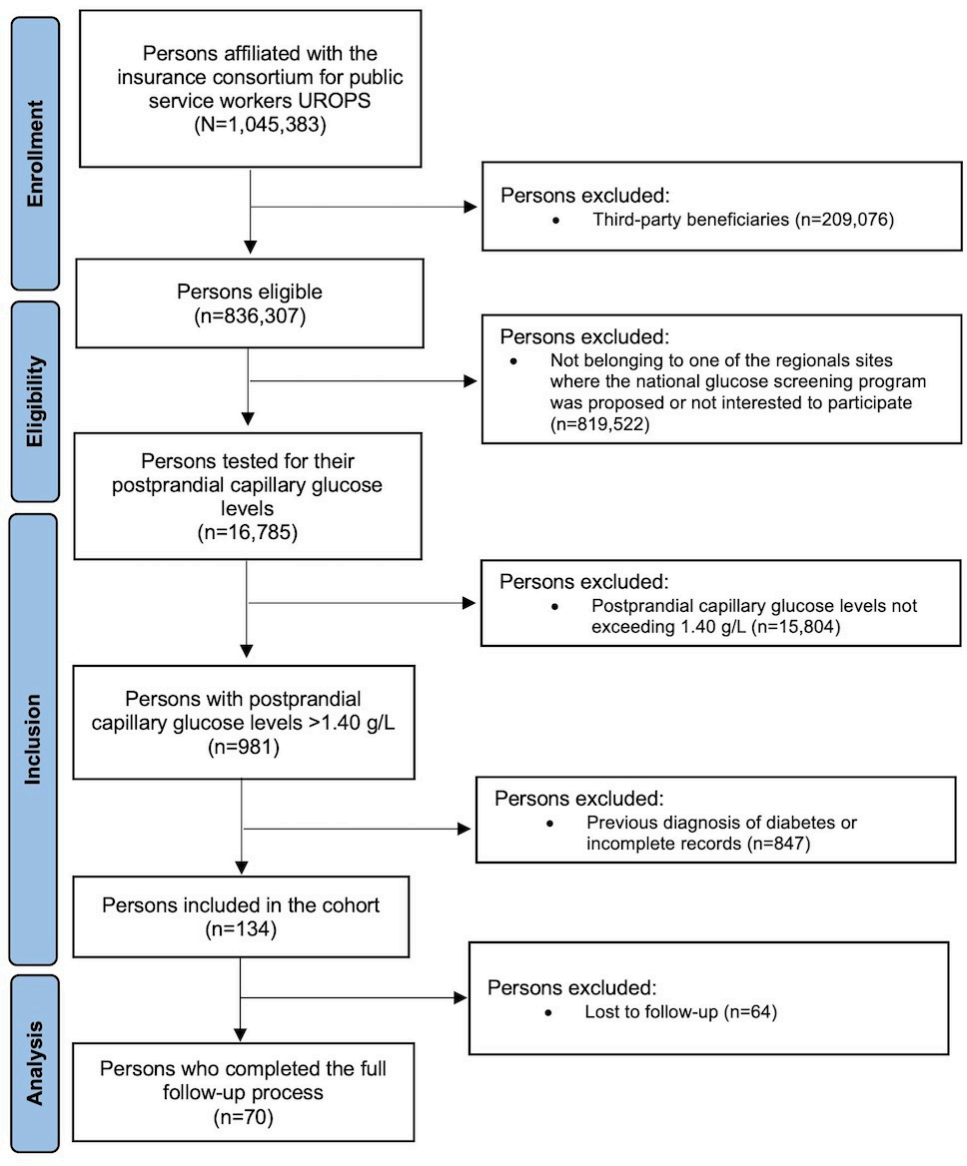
Flowchart of the study. UROPS: *Union Prévention Santé pour la Fonction publique*.

After excluding individuals with a previous diagnosis of diabetes and those with incomplete records, 134 participants were included in the final analytic cohort (Figure 1). Of these, 70 (52.2%) participants completed the full follow-up process, including physician consultation and confirmatory laboratory testing. The remaining 64 (47.8%) participants were lost to follow-up despite repeated attempts to contact them. No adverse events or withdrawals due to data privacy concerns were reported.

This corresponds to a follow-up completion rate of 52.2% (70/134) and an overall yield of 1 newly confirmed diabetes case for every 15 participants followed after abnormal screening.

### Characteristics of Participants

Among the 134 participants, 78 (59.5%) were women and 53 (40.5%) were men. The mean age was 51.3 (SD 8.9) years, with the majority (n=94, 71.8%) of the participants aged between 40 and 59 years.

On the basis of self-reported anthropometric data, 37.6% (n=50) were overweight and 25.6% (n=34) were obese**,** while only 2.3% (n=3) were underweight. Most participants were nonsmokers (n=109, 84.5%) and reported no regular physical activity (n=77, 61.6%; defined as <30 min of physical activity per d). A family history of diabetes was reported by 63.2% (n=84) of participants, and 15.2% (n=20) reported a parental history of myocardial infarction. Personal history of cardiovascular disease was rare (heart attack: n=2, 1.5%; stroke: n=9, 7.2%). Sociodemographic, lifestyle, and cardiometabolic characteristics of participants are represented in Table 1.

**Table 1.** Sociodemographic, lifestyle, and cardiometabolic characteristics of participants included in the observational cohort (N=134).

Variables	Participants, n (%)
Sex (n=131)	
Male	53 (40.5)
Female	78 (59.5)
Age group (years; n=131)	
<30	3 (2.3)
30‐39	10 (7.6)
40‐49	46 (35.1)
50‐59	48 (36.6)
>60	24 (18.3)
BMI (kg/m²; n=133)	
Underweight (<18.5)	3 (2.3)
Normal (18.5-24.9)	46 (34.6)
Overweight (25-29.9)	50 (37.6)
Obese (≥30.0)	34 (25.6)
Tobacco use (n=129)	
No	109 (84.5)
Yes	20 (15.5)
30 minutes of physical activity per day (n=125)	
No	77 (61.6)
Yes	48 (38.4)
History of myocardial infarction in father or mother (n=132)	
No	112 (84.8)
Yes	20 (15.2)
Personal history of heart attack (n=130)	
No	128 (98.5)
Yes	2 (1.5)
Family history of stroke in individuals aged <45 years (n=133)	
No	49 (36.8)
Yes	84 (63.2)
Personal history of stroke (n=125)	
No	116 (92.8)
Yes	9 (7.2)
Family history of diabetes (n=133)	
No	49 (36.8)
Yes	84 (63.2)
Family history of obesity (n=133)	
No	34 (25.6)
Yes	99 (74.4)

### Follow-Up and Diagnostic Outcomes

Among the 70 participants who completed medical follow-up after abnormal glucose screening, 9 (12.9%) received a physician-confirmed diagnosis of T2D.

Confirmed T2D cases were predominantly male (n=7, 78%), and most presented with a BMI more than 25 kg/m² (n=8, 89%). Compared with nondiabetic participants, those with confirmed diabetes were more frequently men (*P*=.04) and more likely to be overweight or obese (*P*=.04).

A trend toward higher physical inactivity was observed among confirmed diabetics, although this difference did not reach statistical significance.

No significant associations were found with age group or family history of diabetes.

## Discussion

### Summary of Key Findings

This study demonstrates that a systematic glucose screening program implemented within the French public sector can effectively identify individuals with previously undiagnosed T2D or at high metabolic and cardiometabolic risk. Among participants with elevated postprandial glucose, more than half (70/134, 52.2%) completed medical follow-up and 12.9% (n=9) were confirmed as new T2D cases, corresponding approximately to 1 newly diagnosed case for every 8 individuals followed after an abnormal glucose test. These findings support the feasibility and potential impact of structured workplace-based screening for diabetes prevention [16,17].

### Comparison With Prior Work

The proportion of newly diagnosed diabetes observed in this study is consistent with results from large population-based screenings in Europe and North America, where detection rates range from 8% to 15% among individuals with abnormal glucose levels [18-21]. Similarly, national surveys in France have shown that approximately 25% of individuals with diabetes remain undiagnosed, a proportion consistent with international estimates [8,22], confirming the magnitude of unrecognized disease in the general population. Our results also align with the National Health Service health check experience in the United Kingdom, which demonstrated improved detection of diabetes and cardiovascular risk factors, together with reduced incidence of multimorbidity [23,24]. Digital and pharmacist-delivered health checks have recently shown comparable effectiveness and acceptable cost-effectiveness ratios (<£10,000 [US $ 13,445] per quality-adjusted life year) [25].

At the global level, underdiagnosis remains a major concern. The Global Diabetes Cascade of Care (2000‐2023) estimated that in 2023 only 55.8% of individuals with diabetes were diagnosed, and merely 21.2% achieved optimal glycemic control [5]. The Centers for Disease Control and Prevention reports similar findings in the United States (approximately 23% undiagnosed adults with diabetes) [6]. The observation that newly confirmed cases in our study were predominantly male mirrors broader evidence showing that men develop diabetes at a younger age and lower BMI than women [26], highlighting the relevance of occupational settings to reach high-risk male populations.

Undetected or poorly controlled diabetes substantially increases the risk of micro and macrovascular complications as well as premature mortality. Large meta-analyses show a 2- to 6-fold higher risk of cardiovascular disease and death in people with diabetes [27,28]. Early diagnosis and intervention are therefore critical. Evidence from the UK Prospective Diabetes Study and later legacy-effect analyses demonstrated that intensive glycemic control initiated early in the disease course yields durable reductions—between 12% and 32%—in complications and mortality [29,30]. These benefits support international guidelines such as the American Diabetes Association (2025), which emphasize confirmatory testing and early management following abnormal screening results [31].

The French public service, employing nearly 5.7 million civil servants—approximately 1 in 5 workers nationwide—offers a uniquely suitable context for preventive interventions [32]. Its organizational stability and national coverage allow standardized screening and long-term follow-up in collaboration with occupational health and mutual insurance networks. Implementing systematic screening and structured follow-up in occupational or community pharmacy settings can help reduce undiagnosed diabetes, improve early management, and reduce complications. Recent studies highlight that integrating strategies such as pharmacist-led testing, digital invitations, automated reminders, and hybrid pharmacy delivery models improves efficiency, uptake, and glycemic outcomes [33,34].

The use of the 1.40 g/L (7.8 mmol/L) postprandial glucose threshold reflects the reference applied in French occupational health screening to identify potential postprandial hyperglycemia requiring confirmatory testing. Although this threshold is nationally defined rather than internationally standardized, extensive international evidence supports the broader principle underlying its use. Elevated postprandial or postload glucose—particularly the 1-hour oral glucose tolerance test measurement—is a strong predictor of future T2D and cardiometabolic complications. A large meta-analysis including more than 35,000 participants showed that 1-hour plasma glucose has high diagnostic accuracy for detecting dysglycemia and early diabetes [35]. Prospective cohort studies have further demonstrated that a 1-hour oral glucose tolerance test value of 155 mg/dL (approximately 8.6 mmol/L) or greater significantly predicts incident diabetes, even among individuals with normal glucose tolerance [36,37]. Elevated 1-hour glucose has also been associated with adverse cardiometabolic profiles [38] and with increased long-term cardiovascular and all-cause mortality [39]. Together, these findings support the rationale for including postprandial glucose assessment in large-scale screening strategies, even though operational thresholds such as 1.40 g/L differ from those used in oral glucose tolerance test–based research protocols.

### Limitations

Several limitations should nevertheless be acknowledged. The retrospective design limits causal inference and may introduce selection bias, as only participants with abnormal glucose levels were included. The follow-up rate (70/134, 52.2%) could have led to an underestimation of the true prevalence of undiagnosed diabetes. No significant differences in age, sex, or BMI were observed between participants who completed follow-up and those lost to follow-up. Nevertheless, because 47.8% (n=64) of participants did not complete the confirmatory evaluation, the possibility of unmeasured differences contributing to follow-up attrition cannot be excluded. Some variables (physical activity, smoking, and anthropometrics) were self-reported and subject to recall bias. Given the retrospective nature of this study, the associations observed cannot be interpreted as causal and should be viewed as descriptive and hypothesis generating.

These results, consistent with current international evidence, reinforce the need to incorporate structured workplace screening and early-detection strategies into national prevention frameworks to reduce the growing burden of diabetes and its complications. Future research should include larger prospective cohorts or randomized screening interventions to confirm the causal impact of workplace-based glucose testing on early diagnosis, engagement in follow-up care, and long-term cardiometabolic outcomes. Such designs would help validate and quantify the true effectiveness of screening pathways implemented in occupational settings.

### Conclusions

This study demonstrates that systematic glucose screening in the French public sector is a feasible and effective way to identify individuals with undiagnosed T2D and individuals at high metabolic risk. With one new case detected for every 15 individuals followed, this program confirms the value of workplace-based initiatives for early diagnosis and prevention.

Integrating such structured screening into occupational health frameworks could significantly reduce the burden of undiagnosed diabetes and its complications. Expanding these strategies—through digital tools, pharmacy networks, or other community settings—would further enhance reach and long-term impact on public health.

## Supplementary material

10.2196/87695 Checklist 1 STROBE checklist.

## Abbreviations

CNIL: *Commission Nationale de l’Informatique et des Libertés*
CONSTANCES: *Cohorte Nationale de Sujets Adultes Nouveaux Constituée pour une Évaluation Systématique de la Santé* (National Cohort of New Adult Subjects Formed for Systematic Health Assessment)
ESTEBAN: *Étude de santé sur l'environnement, la biosurveillance, l'activité physique et la nutrition* (health study on the environment, biomonitoring, physical activity, and nutrition)
STROBE: Strengthening the Reporting of Observational Studies in Epidemiology
T2D: type 2 diabetes
UROPS: *Union Prévention Santé pour la Fonction publique*
